# Beyond Abdominal Pain: Decoding the Mysteries of Nutcracker Syndrome

**DOI:** 10.1155/2024/8702202

**Published:** 2024-07-25

**Authors:** Usamah Al-Anbagi, Abdulrahman Saad, Abdulqadir J. Nashwan

**Affiliations:** ^1^ Medicine Department Hazm Mebaireek General Hospital Hamad Medical Corporation (HMC), Doha, Qatar; ^2^ Public Health Department Ministry of Public Health, Doha, Qatar; ^3^ Nursing Department Hazm Mebaireek General Hospital Hamad Medical Corporation (HMC), Doha, Qatar

**Keywords:** abdominal pain, aorta, CT scan, endovascular procedures, left renal vein (LRV), nutcracker syndrome (NCS), superior mesenteric artery (SMA)

## Abstract

Nutcracker syndrome (NCS), a rare but impactful vascular condition, emerges from the compression of the left renal vein by adjacent major arteries, leading to a diverse array of symptoms such as hematuria, flank pain, and renal challenges. Highlighting the case of a 30-year-old male with an atypical presentation of NCS, this report explores the diagnostic complexities arising from its varied presentations and therapeutic options. It emphasizes the critical role of computed tomography (CT) in unveiling the underlying vascular constriction. Through this lens, the case underscores the necessity of considering NCS in the differential diagnosis of abdominal pain, advocating for a prompt and accurate diagnosis to guide effective management strategies, ranging from conservative approaches to surgical intervention. This stresses the importance of heightened awareness and ongoing research for optimizing patient outcomes in the face of this elusive condition.

## 1. Introduction

Nutcracker Syndrome (NCS) is a rare vascular disorder characterized by compression of the left renal vein between the abdominal aorta and the superior mesenteric artery (SMA) [1]. This compression can lead to a spectrum of clinical manifestations, including hematuria, flank pain, varicocele, and renal impairment [1–3]. However, due to its rarity and varied clinical presentation, diagnosing NCS can pose significant challenges, necessitating a high index of suspicion among clinicians. Early and accurate diagnosis is pivotal in initiating tailored management strategies to alleviate symptoms and prevent complications.

The reported prevalence of NCS initially skewed towards females, but subsequent studies have revealed an equal distribution across genders [4]. Venous reflux is central to the typical clinical presentation, characterized by left flank pain and chronic abdominal discomfort [5]. In women, this syndrome may lead to pelvic congestion syndrome, while in men, it can manifest similarly and is recognized as one of the contributing factors to varicocele formation [4–6].

## 2. Case Presentation

A 30-year-old male presented with a history of moderate abdominal and back pain, predominantly localized to the left lumbar region, persisting for the past year. The pain began suddenly and progressively worsened over the course of 1 day, primarily affecting the left lumbar region but also felt in the left flank. It was described as mild to moderate pain 3–4/10, nonradiating, with no specific character or clear aggravating or relieving factors noted.

The patient denied any associated symptoms of nausea, vomiting, or changes in bowel habits. He reported a history of unintentional weight loss of around 10 kg over the last year, which he attributed to a reduction in appetite, and mentioned occasional feelings of abdominal fullness. He reported no previous medical history except for recurrent episodes of similar pain for which he did not seek medical attention. He denied experiencing hematuria or testicular pain, and there was no family history of relevant medical conditions.

General examination revealed vital signs within normal limits, while the systemic examination was unremarkable except for tenderness noted upon deep palpation of the left lumbar and flank areas. There were no signs of peritoneal irritation or tenderness over the renal angle.

Laboratory investigations yielded normal results, including renal function tests, general urine analysis (negative for proteinuria or hematuria), and other routine blood investigations (Table 1).

**Figure 1:**
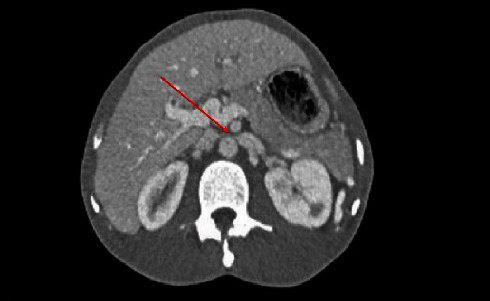
CT scan reveals a dilated left renal vein entrapped between the SMA and aorta with an arrow pointing to the compression area. CT, computed tomography. SMA, superior mesenteric artery.

**Figure 2:**
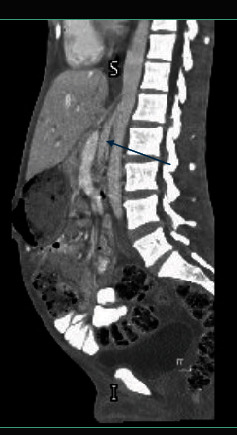
CT scan sagittal reconstruction demonstrates a narrow aortomesenteric with compressed left renal vein.

Further evaluation via computed tomography (CT) imaging revealed features consistent with NCS. The images demonstrated compression of the left renal vein below the SMA origin, accompanied by vein dilatation and splenorenal shunting/collaterals (Figures 1 and 2). Additionally, it clearly ruled out any duodenal or bowel compression.

The patient has been treated conservatively, stabilized with complete resolution of the pain, and discharged safely. He has been referred to the vascular surgery clinic for follow-up.

## 3. Discussion

NCS presents a diagnostic challenge due to its variable clinical manifestations and rarity [1]. Our case underscores the importance of considering NCS in patients with suggestive symptoms like loin pain [1–3].

NCS is clinically divided into two categories: typical and atypical presentations [7]. The typical form manifests with urological symptoms such as gross hematuria and orthostatic proteinuria, often accompanied by flank pain. Conversely, the atypical presentation encompasses nonurologic symptoms, including fatigue, orthostatic intolerance, dysmenorrhea, and dyspareunia in women, while in men, it can lead to varicocele formation [7].

The diagnosis of NCS is typically confirmed through clinical evaluation and imaging studies, with CT scanning serving as the gold standard. Clinical symptoms such as hematuria, flank pain, and varicocele guide suspicion, while characteristic imaging findings of left renal vein compression between the abdominal aorta and SMA, often visualized as the “beak sign” on CT scans, provide definitive evidence. Additional imaging modalities such as ultrasound and magnetic resonance imaging (MRI) may also aid in diagnosis. Laboratory tests, including urinalysis, help to assess renal function and rule out other potential causes of symptoms. Collaboration among healthcare professionals is essential to ensure accurate diagnosis and appropriate management of NCS.

The treatment approach for NCS varies depending on the severity of the symptoms and the presence of complications. Conservative measures, including observation and symptomatic treatment, may suffice for some cases, while others may require more invasive interventions such as surgical or endovascular procedures.

Surgical intervention for NCS typically involves procedures to relieve the left renal vein compression [8]. Left renal vein transposition involves rerouting the vein below the SMA, while renal vein stenting entails inserting a stent to keep the vein open. In severe cases, renal autotransplantation may be considered [9]. These procedures are aimed at restoring normal blood flow, alleviating symptoms such as hematuria and flank pain, and preventing complications [10]. The choice of surgical approach depends on various factors, including the severity of symptoms and anatomical considerations, with decisions made in consultation with a multidisciplinary team of healthcare providers specialized in vascular surgery and interventional radiology [8–10].

The long-term prognosis of patients with NCS depends on various factors, including the severity of venous compression, the effectiveness of treatment, and the presence of any underlying renal dysfunction [10]. Continued research and clinical vigilance are essential for further elucidating the pathophysiology of NCS and refining treatment algorithms to improve outcomes for affected individuals.

## 4. Conclusions

In conclusion, our case underscores the importance of considering NCS in patients with abdominal pain. Timely diagnosis using imaging modalities like CT scans is crucial for implementing appropriate management strategies tailored to the patient's needs. While conservative methods suffice for some cases of NCS, others may require surgical or endovascular interventions to alleviate symptoms and prevent complications.

## Figures and Tables

**Table 1:** Laboratory results.

**Parameters**	**Result**	**Reference values**
Total leukocytes	3.7	(6.2 × 10^3^/uL)
Hemoglobin	14.2	(13–17 gm/dL)
Serum potassium K (mmol/L)	3.8	(3.5–5.3)
Serum sodium (mmol/L)	140	(133–146)
Serum calcium (mmol/L)		(2.2–2.6)
Serum urea (mmol/L)	10.1	(2.5–7.8)
Serum creatinine (umol/L)	96	(62–106)
Serum albumin (gm/L)	40	(35–50)
Serum total protein (gm/L)	76	(60–80)
Lactate (mmol/L)	1.4	(0.5–2.2)
AST (IU/L)	95	(0–41)
ALT (IU/L)	114	(0–41)
Alkaline phosphatase (U/L)	58	(40–129)
Serum total bilirubin (mg/dl)	9	(0–21)
Serum bicarbonate (mmol/L)	25.8	(22–29)

Abbreviations: AST, aspartate aminotransferase; ALT, alanine aminotransferase.

## Data Availability

The data that support the findings of this study are available from the corresponding author upon reasonable request.
